# GLPG1205, a GPR84 Modulator: Safety, Pharmacokinetics, and Pharmacodynamics in Healthy Subjects

**DOI:** 10.1002/cpdd.955

**Published:** 2021-05-07

**Authors:** Helen Timmis, Tim Van Kaem, Julie Desrivot, Sonia Dupont, Luc Meuleners, Johan Beetens, Eric Helmer, Eva Santermans, Silke Huettner

**Affiliations:** ^1^ Galapagos Biotech Limited Cambridge UK; ^2^ Galapagos Mechelen Belgium; ^3^ Galapagos Romainville France

**Keywords:** first‐in‐human, GLPG1205, pharmacodynamics, pharmacokinetics, safety

## Abstract

GLPG1205 is a modulator of GPR84, a G‐protein–coupled receptor reported to be associated with several diseases. Safety, tolerability, pharmacokinetics, and pharmacodynamics of GLPG1205 in healthy subjects were evaluated in 2 randomized, double‐blind, placebo‐controlled, single‐site, phase 1 studies. In study 1, 16 (aged 21‐48 years) and 24 (24‐50 years) healthy men received single doses of GLPG1205 10 to 800 mg, and GLPG1205 50, 100, or 200 mg once daily for 14 days, respectively, or placebo. Study 2 evaluated the effect of aging on GLPG1205 pharmacokinetics: 24 healthy men (aged 37–83 years), weight‐matched into 3 age cohorts (65‐74, ≥75, and 18‐50 years), received GLPG1205 50 mg or placebo once daily for 14 days; an open‐label part of this study evaluated a GLPG1205 250‐mg loading dose followed by 50 mg once daily for 13 days in 8 healthy men (aged 68‐74 years). Single (up to 800 mg) and multiple (maximum tolerated dose 100 mg once daily) GLPG1205 doses had favorable safety and tolerability profiles. After single administration of GLPG1205, median time to occurrence of maximum observed plasma concentration and arithmetic mean apparent terminal half‐life ranged from 2.0 to 4.0 and from 30.1 to 140 hours, respectively. Age did not affect GLPG1205 exposure. GPR84 receptor occupancy with GLPG1205 vs placebo confirmed target engagement. These results support further clinical development of GLPG1205.

GPR84 is a G‐protein–coupled receptor activated by medium‐chain fatty acids, which is primarily expressed on innate immune cells such as polymorphonuclear leukocytes, monocytes, and macrophages.^1^, ^2^, ^3^ GPR84 is also expressed in a number of major organs (eg, brain, heart, kidney, liver, and lung).^1^, ^2^

GPR84 has been identified as a mediator in inflammatory, metabolic, and fibrotic disease progression.^1^, ^2^, ^3^, ^4^ GPR84 amplifies interleukin (IL)‐8, IL‐12, and tumor necrosis factor‐α production, and is classified as a proinflammatory receptor.^2^, ^3^ In addition, activation of GPR84 is thought to diminish release of adiponectin, a protein hormone that protects against insulin resistance/diabetes,^5^ and GPR84 expression levels are increased by hyperglycemia.^1^, ^4^, ^6^ Furthermore, GPR84 activation may promote fibrosis.^1^ Therefore, agents that inhibit GPR84 may delay or prevent inflammatory, metabolic, or fibrotic disease progression.^1^, ^2^

GLPG1205 (9‐cyclopropylethynyl‐2‐((S)‐1‐[1,4]dioxan‐2‐ylmethoxy)‐6,7‐dihydropyrimido[6,1‐a]isoquinolin‐4‐one; compound code G321605; Figure 1) is a GPR84 modulator that has been shown to selectively and potently inhibit GPR84 activation in GPR84‐overexpressing human embryonic kidney cells, as well as GPR84‐induced neutrophil migration.^7^, ^8^ The PK profile of GLPG1205 has been assessed in 3 animal species (mouse, rat, and dog), and showed an oral absolute exposure of ≥68% and an apparent terminal half‐life of 1.3 to 2.0 hours.^8^ Plasma protein binding was high (≥ 92%) in human and animals.^8^ GLPG1205 exposure increased dose‐proportionally up to doses of 100 and 30 mg/kg/d in rats and monkeys, respectively.^8^ The main enzymes involved in GLPG1205 metabolism are cytochrome P450 (CYP) 3A4 and CYP2C19. In vitro interaction studies of GLPG1205 with CYP enzymes showed weak inhibition of CYP2B6, CYP2C8, CYP2C9, and CYP2C19 enzymes and weak induction of CYP1A2 (data on file at Galapagos). A clinical drug‐drug interaction study demonstrated that GLP1205 100 mg once daily did not affect the exposure of CYP1A2, CYP2C9, or CYP2C19 enzymes to a clinically relevant extent in healthy male subjects (data on file at Galapagos).^8^, ^9^

**Figure 1:**
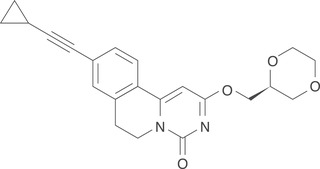
Chemical structure of G321605 (the compound code for GLPG1205).

This article presents data from the first‐in‐human study of GLPG1205, which aimed to evaluate the safety, tolerability, pharmacokinetics (PK), and pharmacodynamics (PD) of single and multiple ascending doses of GLPG1205 vs placebo in healthy men. This article also includes findings from a second study that evaluated the safety, tolerability, and PK of multiple doses of GLPG1205 in healthy men of different ages and of a loading dose followed by once‐daily dosing of GLPG1205.

## Methods

### Study Designs

Aspects of study design for the first‐in‐human study (study 1) and the effect of aging and loading dose study (study 2) are summarized in Table 1. Both studies were conducted at a single investigational site (SGS Life Science Services, Mechelen, Belgium) and in accordance with the Declaration of Helsinki and Good Clinical Practice guidelines, and were approved by an independent ethics committee at the site and the Federal Agency for Medicines and Health Products (Belgium). All subjects in both studies provided written informed consent before enrollment.

**Table 1:** Summary of Study Designs for the First‐in‐Human and Effect of Aging and Loading Dose Studies

	First‐in‐Human Study (Study 1)	Effect of Aging and Loading Dose Study (Study 2)
Phase	1	1
Type	Randomized, double‐blind, placebo‐controlled study of GLPG1205 (part 1: SAD; part 2: MAD)	Randomized, double‐blind, placebo‐controlled study of multiple doses of GLPG1205 (part 1), and an open‐label evaluation of a loading dose followed by multiple doses of GLPG1205 (part 2)
Clinicaltrials.gov number	NCT01887106	NCT03102567
Primary objective(s)	To evaluate the safety and tolerability of SAD and MAD of GLPG1205 in healthy subjects	To evaluate the safety and tolerability of multiple doses of GLPG1205 in healthy elderly (aged ≥65 y) male subjects compared with younger (aged 18‐50 y) male subjects, to assess the effect of aging on the PK of multiple GLPG1205 doses, and to characterize the PK profile of multiple GLPG1205 doses when starting with a loading dose
Select secondary objectives	To evaluate the PK and PD of GLPG1205 after single and multiple administrations	
Key inclusion criteria	Male; aged 18‐50 y, inclusive; BMI, 18‐30 kg/m^2^, inclusive; judged to be in good health; discontinued any medications^a^ at least 2 weeks before first study drug administration and did not take any medications during the study; no alcohol consumption during the study; a nonsmoker; and a negative urine drug screen	Male; aged ≥18 y; BMI, 18‐30 kg/m^2^, inclusive; body weight, 60‐90 kg, inclusive (cohort A only); judged to be in good health; discontinued any medications^a^, ^b^ at least 3 wk (or 5 half‐lives of the drug, whichever was longer) before first study drug administration; no alcohol consumption during the study; and a creatinine clearance (estimated by Cockcroft‐Gault equation) >80 mL/min for subjects aged up to 50 y in cohort C, and >60 mL/min for subjects aged ≥65 y in cohorts A, B, and D
Randomization and blinding	Randomization ensured a 3:1 allocation to GLPG1205 treatment or placebo in each single‐dose cohort (A and B) and in each multiple‐dose cohort (C, D, and E). In addition, subjects in cohorts A and B were randomized to 1 of 4 treatment sequences The subjects, clinical study staff, and sponsor were blinded to treatment	In part 1, subjects in cohort A were randomized 3:1 to GLPG1205 or placebo; subjects in Cohorts B and C were matched by body weight 1:1 to the subjects in cohort A and were assigned to GLPG1205 or placebo accordingly. The subjects, clinical study staff, and sponsor were blinded to treatment in part 1 Part 2 was open‐label, single‐arm

BMI, body mass index; MAD, multiple ascending doses; PD, pharmacodynamics; PK, pharmacokinetics; SAD, single ascending doses.

^a^ Excluding occasional acetaminophen (maximum dose of 2 g/d and a maximum of 10 g/2 wk).

^b^ Medication for cardiac protection, such as low‐dose aspirin, or for chronic stable conditions was allowed at the discretion of the investigator and had to continue unchanged throughout the study.

#### Study 1

GLPG1205 or matching placebo were administered as an oral nanosuspension in the morning in a fed condition as an outpatient. For both the single ascending doses (SADs) and multiple ascending doses (MADs) parts of the study, subjects attended a screening visit (21 to 2 days before first study drug administration) and a follow‐up visit (7 to 10 days after the last dose).

The SAD part of the study comprised 16 healthy male subjects in 2 alternating cohorts (A and B, n = 8 each). Cohort A received GLPG1205 10, 90, 400, and 800 mg or matching placebo, and cohort B received GLPG1205 30, 200, and 600 mg or matching placebo. Subjects were randomly assigned to receive GLPG1205 or matching placebo in a 3:1 ratio once at the beginning of the study. In addition, subjects in cohorts A and B were randomized to a treatment sequence. Each subject, in either cohort A or cohort B, had an enforced interval of at least 6 days between dosages. An interval of at least 3 days was enforced between 2 dose levels (between cohort A and B). Subjects were kept in‐house from the evening of day –1 to  ≈26 hours after dosing (morning of day 2).

In the MAD part of study 1, 24 healthy male subjects in 3 cohorts (C, D, and E; n = 8 each) each received GLPG1205 or matching placebo once daily for 14 days. Cohorts C, D, and E received GLPG1205 50 mg once daily or matching placebo, GLPG1205 100 mg once daily or matching placebo, and GLPG1205 200 mg once daily or matching placebo, respectively. Within a cohort, subjects were randomized to receive GLPG1205 or matching placebo in a 3:1 ratio. An interval of at least 6 days was enforced between cohorts. Subjects were kept in‐house from the evening of day –1 until ≈26 hours after first dosing (morning of day 2), and from the evening of day 13 to the morning of day 15. Administration of the study drug was performed daily at the clinical pharmacology unit.

#### Study 2

During study 2, GLPG1205 50 mg or matching placebo was administered as capsules in the morning 30 minutes after the start of a standard breakfast. Subjects were kept in‐house from the evening of day –1 to ≈26 hours after the first dose (day 2), and from the evening of day 13 until day 15. Administration of the study drug was performed daily at the clinical pharmacology unit. Subjects returned for a follow‐up visit at day 35.

In part 1, 24 healthy male subjects were matched into 3 cohorts based on body weight: Cohort A comprised 8 subjects aged 65–74 years, inclusive; cohort B comprised 8 subjects aged ≥75 years (1:1 weight matched with cohort A subjects [± 5 kg]); and cohort C comprised 8 subjects aged 18 to 50 years, inclusive (1:1 weight matched with cohort A subjects [± 5 kg]). All cohorts received GLPG1205 50 mg once daily or matching placebo, in a 3:1 ratio, for 14 days. In the open‐label second part of study 2, 8 subjects (cohort D) aged 65 to 74 years, inclusive, were included to characterize the PK profile after a loading dose of GLPG1205 250 mg on day 1 followed by multiple doses of GLPG1205 50 mg once daily from day 2 to day 14.

### Study Participants

Key inclusion criteria for studies 1 and 2 are shown in Table 1 and exclusion criteria for both studies can be found in Table S1. Male subjects aged 18 to 50 years were considered an appropriate and homogeneous group for use in these studies. In study 2, male subjects aged >18 years were considered appropriate for the study, which included a cohort of subjects aged ≥75 years. In both studies, subjects were required to be otherwise healthy and subjects with any clinically significant illness in the 12 weeks before the first intake of the study drug were excluded. Subjects were required not to consume alcohol or large amounts of caffeine, or take other medications, during both studies.

### Safety and Tolerability Assessments

Safety and tolerability were assessed on the basis of adverse events (AEs), which were monitored throughout both studies. Additional safety assessments included vital signs (including supine [and standing in study 2] heart rate, systolic and diastolic blood pressure, and oral body temperature), 12‐lead electrocardiogram (ECG), clinical laboratory tests (hematology, coagulation [study 2 only], serum/plasma chemistry, urinalysis, urine drug screen, serology, and alcohol breath test), and a comprehensive physical examination. In the SAD part of study 1, clinical laboratory tests, physical examination, and vital signs were assessed at the screening visit, at the time of dosing (0 hours after dose), 24 hours after dosing and at follow‐up (7 to 10 days after the final dose). Vital signs were additionally observed 2 hours after dosing, and the 12‐lead ECG was additionally completed at 1, 2, 6, 8, and 12 hours after dosing. In the MAD part of study 1, all additional safety assessments were performed at screening; days 1, 2, 8, 14, and 15; and at follow‐up. In study 2, additional safety assessments were performed at screening (between 21 and 2 days before the first study drug administration); days 1, 2, 5, 10, 14, 15, and 20 (clinical laboratory tests were not performed on day 20); at early discontinuation; and at follow‐up. For study 2, renal function tests (measurement of estimated creatinine clearance rate using the Cockcroft‐Gault equation) were also included in the clinical laboratory tests for safety assessment.

### Pharmacokinetic Assessments

In the SAD part of study 1, blood samples (2 mL) for PK assessments were obtained before dosing and at multiple time points on the day of study drug administration (before dosing and 0.5, 1, 2, 4, 6, 8, and 12 hours after dosing) and at 24, 48, and 72 hours after dosing. The predose sample for the next dose level was also used in PK analysis (168 hours after dosing). For doses 400 to 800 mg, due to interim PK sample analysis demonstrating that the half‐life of GLPG1205 was longer than initially predicted, the 0.5‐hour postdose sample on the day of study drug administration was replaced by a sample at 72 hours after dosing. In the MAD part of study 1, blood samples (2 mL) were obtained on days 1, 2, 4, 5, 6, 8, 10, 12, 14, 15, 16, 17, and 18 and at follow‐up (approximately 7 to 10 days after the last dose). These PK blood samples were taken after dosing (1, 2, 4, 6, 8, 12, and 24 hours) on day 1; before dosing on days 4, 6, 8, and 10; and after dosing (1, 2, 4, 6, 8, 12, 24, 72, and 96 hours) on day 14. The following additional PK blood samples were also taken: cohort C, before dosing on day 5; and cohorts D and E, before dosing on day 12. Urine samples were obtained from fractions collected over 6‐ or 12‐hour periods after dosing on days –1, 1, 2, 13, 14, and 15; samples obtained on days –1 and 13 were for cytochrome P450 (CYP) induction analysis only. In study 2, blood samples (2 mL) were obtained on days 1, 2, 5, 7, 10, 14, 15, 17, and 20 and at follow‐up (approximately 21 days after end of study medication administration). Blood samples were taken before dosing and at 1, 2, 4, 6, 8, 12, and 24 hours after drug administration on days 1 and 14 and before dosing on other sampling days.

Blood samples were immediately chilled (ice bath), and the plasma was separated by centrifugation (4°C for 10 minutes at ≈1500 *g*) within 30 minutes of blood collection.

GLPG1205 plasma concentrations were determined using a validated liquid chromatography with tandem mass spectrometry method. After a protein precipitation with methanol, a chromatographic separation was performed on a Kinetex C18 column (50×3.0 mm, 2.6 μm; Phenomenex, Torrance, California) set at 40°C by using a Nexera high‐performance liquid chromatography (HPLC) system (Shimadzu, Kyoto, Japan) or an Infinity HPLC system (Agilent Technologies, Diegem, Belgium) in isocratic elution mode. A QTRAP6500, QTRAP4000, or API4000 mass spectrometer (AB Sciex, Nieuwerkerk aan den Ijssel, The Netherlands) equipped with a TurboIonSpray probe operated in the multiple reaction monitoring in positive mode was used for quantification. The calibration curves in plasma were linear over the range of 1 to 1000 ng/mL with 1/x^2^ as weighting factor. The limit of quantification of the assay in the plasma samples was set at 1 ng/mL.

GLPG1205 concentrations in urine fractions were determined by using a qualified liquid chromatography with tandem mass spectrometry method derived from the plasma method. Chromatographic separation was performed on the product obtained after extraction with methanol by using a Kinetex C18 column (50×3.0 mm, 2.6 μm; Phenomenex) set at 40°C by using an 1100 series HPLC system (Agilent) in isocratic elution mode. An API4000 mass spectrometer (AB Sciex) equipped with a TurboIonSpray probe operated in the multiple reaction monitoring in positive mode was used for quantification. The calibration curves in urine were linear over the range of 10 to 10 000 ng/mL with 1/x^2^ as weighting factor. The limit of quantification of the assay for the urine samples was set at 10 ng/mL.

PK calculations were performed using Phoenix WinNonlin 6.2 (Pharsight Corporation, Palo Alto, California). PK parameters determined for GLPG1205 (from individual plasma and/or urine concentration‐time profiles where appropriate) included the maximum observed plasma concentration (C_max_); plasma concentration at 24 hours after dosing (C_24h_); average plasma concentration; the time occurrence of C_max_ (t_max_); the area under the plasma concentration–time curve from time 0 to infinity (AUC_0‐inf_) and from time 0 to 24 hours (AUC_0‐24h_); area under the plasma concentration‐time curve over dosing interval (AUC_Τ_); the apparent terminal half‐life (t_1/2,λz_); accumulation ratio (R_ac_); renal clearance; and the cumulative amount of GLPG1205 excreted in urine (Ae) over 24 hours. AUC_0‐inf_ was calculated from the area under the plasma concentration‐time curve from time 0 until the time corresponding with the last observed quantifiable concentration + C_t_/λ_z_, where C_t_ was the last observed quantifiable concentration and λ*_z_* the first‐order terminal rate constant. AUC_0–24h_ and AUC_Τ_ were calculated by the linear‐logarithmic trapezoidal rule. t_1/2,λz_ was calculated from (ln 2)/λ_z_. R_ac_ was calculated as AUC_Τ_ day 14/AUC_Τ_ day 1 (study 1, MAD part only) or AUC_0‐24h_ day 14/ AUC_0‐24h_ day 1 (study 2). Renal clearance was calculated as Ae/AUC, where Ae and AUC were calculated over the same interval (study 1, MAD part only).

The potential of CYP3A4 induction was assessed by means of the ratio of 6β‐OH‐cortisol to cortisol in urine.^10^, ^11^, ^12^, ^13^, ^14^ Cortisol concentrations in urine were determined by using a radioimmunoassay method based on competition between labeled antigens and antigens on the specific sites of the antiserum coated on the tubes. At the end of the incubation period, the liquid in the tubes was removed by aspiration and the radioactivity (^125^I‐cortisol) was measured using a gamma counter (Packard Cobra II auto‐gamma counter; Packard Instrument Co Inc, Meriden, Connecticut). The assays were performed using cortisol radioimmunoassay CT test kits (RADIM, Freiburg im Breisgau, Germany) including calibrator samples ranging from 10 to 800 ng/mL. The calibration equation was computed using Prism (GraphPad Software, La Jolla, California) by plotting the log of cortisol concentrations (ng/mL) vs the logit B/Bo. The best curve was determined by the polynomial second‐order equation. The limit of quantification of the cortisol assay for the urine samples was set at 10 ng/mL.

6β‐OH‐cortisol concentrations in urine were determined by using a 2‐step, quantitative competitive enzyme immunoassay technique. The assay was performed using 6β‐hydroxycortisol kits from Stabiligen (Villers‐lès‐Nancy, France) including calibrator samples ranging from 50 to 1000 pg/mL. The calibration equation was computed using SoftMax Pro software (Molecular Devices, Sunnyvale, California) by plotting the log of 6β‐OH‐cortisol concentrations versus the A/Ao (when A was standard or sample 6β‐OH‐cortisol absorbance and Ao was the standard 0 absorbance). The best line was determined by using the 4‐parameter logistic model. The limit of quantification of the 6β‐OH‐cortisol assay for the urine samples was set at 50 pg/mL.

### Pharmacodynamic Assessments

In study 1, ex vivo GPR84 receptor occupancy by GLPG1205 was determined in blood samples (3 mL) to assess target engagement in a clinical setting. In the SAD part of the study, blood samples were collected on day 1 (before dosing and 0.5, 1, 2, 4, and 8 hours after dosing) and at 24 hours after dosing. In the MAD part of the study, samples were taken on days 1 and 14 (before dosing and 0.5, 1, 2, 4, and 8 hours after dosing) and at 24 hours after dosing on days 2 (ie, before dosing on day 2) and 15. In the SAD part of the study, no samples for PD assessment were taken for GLPG1205 10 mg as no PD effect was expected at this dose based on preclinical pharmacological data. Blood samples were processed as soon as possible or within 4 hours of collection and stored at room temperature without shaking. A binding assay was used to evaluate ex vivo GPR84 receptor occupancy by GLPG1205 by displacement of a radiolabeled form of a GPR84 modulator [^3^H]‐G259543.^15^ Blood samples were diluted 1:1 in phosphate buffered saline containing 0.05% bovine serum albumin before incubation of 800 μL of diluted blood per condition with [^3^H]‐G259543 (in duplicate; to determine the total binding potential) or [^3^H]‐G259543 in the presence of an excess of cold G259543 (in duplicate; to determine the nonspecific receptor binding). Red blood cells were then lysed and the cell suspension filtrated onto a filter plate and washed before measurement of radioactivity. The percent of inhibition of tritiated ligand binding by GLPG1205 for a subject (*x*) at each time point (*t*) for each replicate (*i*) was derived as:

$$=100\%\times \left(1-\frac{\text{specific}\;\mathrm{binding}\;\left(x,\;t,\;i\right)}{\mathrm{mean}\;\mathrm{baseline}\;\text{specific}\;\mathrm{binding}\;\left(x\right)}\right)$$

where the specific binding (*x, t, i*) = total binding (*x, t, i*) – mean_j_ [nonspecific binding (*x, t, j*)], and the mean specific binding (*x*) = *mean_t_*_= –40min,–10min_ {*mean_j_* [total binding (*x, t, j*)] – *mean_j_* [nonspecific binding (*x, t, j*)]}.

### Statistical Analysis

In both studies, the safety population comprised all subjects who received at least 1 dose of the study drug and in the PK analysis, all subjects who were exposed to GLPG1205 and who had available and evaluable data were included. The PD analysis population in study 1 comprised the safety population excluding all major protocol violations and the GLPG1205 10‐mg dose (as no PD effect was expected at this dose, based on preclinical pharmacological data); due to the dose adaptation (GLPG1205 200 to 150 mg) in cohort E (see Results section for further details), only day 1 data from this cohort were included in the formal PD analysis. The number of subjects included in each study was expected to give a reasonable precision around the estimates derived for PK and PD evaluations.

In both studies, the safety analysis was descriptive and focused on changes from baseline and treatment‐emergent findings.

#### Study 1

Descriptive statistics were calculated by dose (SAD part), and by dose and day (MAD part), for GLPG1205 plasma concentrations (and urine amounts for the MAD part) and PK parameters. Descriptive statistics were not calculated in the case of very few observations (n < 3), except for arithmetic mean which was presented if n ≥ 2. In the SAD part of the study, dose‐proportionality of the PK parameters was assessed using a mixed‐effects analysis of variance (ANOVA) with cohort and dose as fixed effects, on the following ln‐transformed, dose‐adjusted PK parameters. In the MAD part of the study, dose‐proportionality was assessed using a mixed‐effects ANOVA with day, dose and dose*day interaction as fixed effects, on the following ln‐transformed, dose‐adjusted PK parameters. In case of a significant dose effect, comparison between doses was performed using Tukey's test.

In the MAD part of study 1, time to reach steady state was assessed using GLPG1205 trough plasma concentrations. Time to reach steady state was assessed by visual inspection of the trough plasma concentrations and by using a mixed‐effects ANOVA with day as fixed effect on ln‐transformed GLPG1205 trough plasma concentrations at each dose level. Comparison between days was performed using Tukey's test.

Descriptive statistics were calculated by dose for the urine amounts of 6β‐OH‐cortisol and cortisol, as well as for the 6β‐OH‐cortisol/cortisol ratio on days –1 and 13. The potential induction of CYP3A4 was assessed using a mixed‐effects ANOVA on log‐transformed 6β‐OH‐cortisol/cortisol ratio, with dose, day, and dose*day interaction as fixed effects.

For the PD assessment, raw counts‐per‐minute values were used to calculate the percentage inhibition of the maximal specific binding measured before dosing. The nonspecific binding was subtracted from the total binding to get the specific binding, and then the percentage inhibition compared with the individual baseline calculated. PD data were summarized descriptively per treatment.

#### Study 2

Descriptive statistics were used to present GLPG1205 plasma concentrations and PK parameters stratified by cohort. For cohorts A, B, and C, a mixed model was used to assess significant age or day effects on the PK parameters. In cases of a significant effect, pairwise comparisons were performed using Tukey's test. As t_max_ was a discrete variable dependent on selected blood sampling times, the effect of aging and of day were assessed using nonparametric tests. In the loading dose part of the study, an ANOVA model with cohort (A and D) as fixed effect was used. Point estimates were calculated as the geometric mean of the individual ratios for each parameter for cohort D relative to cohort A, and expressed as a percentage.

Time to reach steady state was assessed for each cohort separately by visual inspection of the trough plasma concentrations on each day and by using a mixed‐effects model with day as fixed effect on ln‐transformed GLPG1205 trough plasma concentrations. Pairwise comparison between days could be performed and corrected for multiple testing (simulation‐based adjusted *P* value) if the overall “day” effect was statistically significant.

## Results

### Study Disposition and Demographics

In study 1, 16 and 24 healthy subjects were randomized in the SAD and MAD parts of the study, respectively (Figure S1). All randomized subjects completed the study; the 3 subjects in the MAD part who discontinued the study drug mainly due to headache performed all remaining study visits per protocol (see Safety and Tolerability section for further details). The study was conducted from June 14 to September 23, 2013. Subjects enrolled in the SAD part of the study were all men (White, n = 15; Black or African American, n = 1) with a median (range) age of 33.0 (21 to 48) years (Table 2A). Subjects enrolled in the MAD part of the study were all White men, with a median (range) age of 38.0 (24 to 50) years (Table 2B).

**Table 2:** Demographics for Healthy Male Subjects in the (A) SAD and (B) MAD Parts of Study 1

A			
	Cohort A (n = 8)	Cohort B (n = 8)	SAD Total (n = 16)
Age, y			
Mean (SE)	38.0 (2.52)	29.3 (3.07)	33.6 (2.23)
Median (range)	38.0 (27‐48)	27.0 (21‐42)	33.0 (21‐48)
Weight, kg			
Mean (SE)	81.0 (4.09)	81.5 (3.41)	81.3 (2.57)
Median (range)	80.5 (68‐102)	81.5 (69‐102)	81.0 (68‐102)
BMI, kg/m^2^			
Mean (SE)	25.3 (1.03)	23.8 (0.59)	24.5 (0.61)
Median (range)	25.0 (22‐29)	23.5 (22‐27)	24.0 (22‐29)
Race, n (%)			
Black or African American	1 (12.5)	0	1 (6.3)
White	7 (87.5)	8 (100.0)	15 (93.8)

B					
	Pooled Placebo (n = 6)	GLPG1205 50 mg Once Daily (n = 6)	GLPG1205 100 mg Once Daily (n = 6)	GLPG1205 200 mg Once Daily (n = 6)	MAD Total (n = 24)
Age, y					
Mean (SE)	33.3 (3.54)	38.7 (3.30)	41.3 (3.27)	42.7 (3.20)	39.0 (1.72)
Median (range)	32.5 (24‐47)	41.0 (28‐47)	42.5 (29‐50)	46.0 (31‐50)	38.0 (24‐50)
Weight, kg					
Mean (SE)	82.3 (5.87)	83.5 (3.12)	84.2 (4.55)	75.2 (5.01)	81.3 (2.34)
Median (range)	83.0 (60‐102)	82.0 (77‐95)	84.0 (72‐99)	73.0 (57‐94)	80.0 (57‐102)
BMI, kg/m^2^					
Mean (SE)	24.0 (1.39)	26.0 (0.82)	25.3 (1.09)	23.7 (0.71)	24.8 (0.52)
Median (range)	24.5 (18‐28)	27.0 (23‐28)	26.5 (22‐28)	23.5 (21‐26)	25.0 (18‐28)
Race, n (%)					
White	6 (100)	6 (100)	6 (100)	6 (100)	24 (100)

BMI, body mass index; MAD, multiple ascending doses; SAD, single ascending doses; SE, standard error.

In study 2, 32 healthy subjects were randomly assigned and received at least 1 dose of the study drug or matching placebo, 24 in the aging part of the study and 8 in the loading dose part (Figure S2). One subject receiving placebo was withdrawn from the study due to an AE (pain in extremity); 31 subjects completed the study. The study was conducted from October 18, 2016, to February 23, 2017. Subjects were all white men, with median (range) ages of 70.0 (37 to 81), 70.5 (67 to 73), 77.0 (75 to 83), and 48.0 (37 to 50) years for the pooled placebo, 65 to 74 years, ≥75 years, and 18 to 50 years groups, respectively in part 1, and a median (range) age of 70.5 (68 to 74) years in part 2 (Table 3).

**Table 3:** Demographics for Healthy Male Subjects in Study 2

	Part 1				Part 2, Open Label
	Pooled Placebo (n = 6)	GLPG1205 50 mg Once Daily, 65‐74 y (n = 6)	GLPG1205 50 mg Once Daily, ≥75 y (n = 6)	GLPG1205 50 mg Once Daily, 18‐50 y (n = 6)	GLPG1205 250‐mg Loading Dose + 50 mg Once Daily, 65‐74 y (n = 8)
Age, y					
Mean (SE)	62.2 (8.17)	70.2 (1.17)	77.7 (1.15)	46.5 (2.01)	70.6 (0.73)
Median (range)	70.0 (37‐81)	70.5 (67‐73)	77.0 (75‐83)	48.0 (37‐50)	70.5 (68‐74)
Weight, kg					
Mean (SE)	78.93 (1.08)	78.83 (2.91)	77.65 (3.48)	78.58 (1.89)	77.81 (3.53)
Median (range)	78.75 (76.0‐83.0)	78.30 (70.9‐88.5)	76.08 (69.8‐92.0)	77.65 (71.8‐84.2)	78.10 (58.3‐90.9)
BMI, kg/m^2^					
Mean (SE)	26.77 (0.65)	25.63 (0.91)	27.08 (0.81)	25.28 (0.66)	25.2 (1.00)
Median (range)	26.95 (24.6‐28.5)	25.45 (23.2‐28.5)	27.25 (24.2‐29.6)	25.05 (23.5‐27.7)	25.15 (20.0‐28.9)
Race, n (%)					
White	6 (100.0)	6 (100.0)	6 (100.0)	6 (100.0)	8 (100.0)

BMI, body mass index; SE, standard error.

### Safety and Tolerability

Across both studies and all dose groups, treatment‐emergent AEs (TEAEs) were reported for 44 subjects following administration of GLPG1205 and for 7 subjects following administration of placebo. Frequently occurring TEAEs following administration of GLPG1205 included headache and nausea (17 and 12 subjects, respectively). No deaths or serious AEs occurred, and no clinically significant trends were observed for ECG and vital signs during both studies.

#### Study 1

In the SAD part of study 1, the most frequently reported TEAE was nausea (observed in 4 subjects), all cases of which were rated mild in intensity and all but 1 were considered at least possibly treatment related. The physical examination did not reveal any clinically relevant abnormalities. One clinically significant laboratory result was observed in 1 subject who had a positive test for chlamydial infection (considered unrelated to treatment). This was reported as a TEAE (urethritis chlamydial infection; for full details on TEAEs see Table S2A).

In the MAD part of study 1, the most frequently reported TEAE was headache (placebo, n = 1; GLPG1205 100 mg once daily, n = 3; GLPG1205 200 mg once daily, n = 4); all cases of headache reported in the GLPG1205 200 mg once daily group (n = 4) were considered at least possibly treatment related. Study drug was withdrawn for 3 of the 4 subjects in the GLPG1205 200‐mg once‐daily groups who experienced a TEAE of headache. In these 3 subjects, TEAEs that led to study drug withdrawal were: headache (n = 3); dehydration, vomiting, fatigue (n = 2 for each); dizziness, diarrhea, decreased appetite, abdominal pain, flatulence, musculoskeletal stiffness, and nausea (n = 1 for each). Based on these observations, the daily dose was lowered from GLPG1205 200 to 150 mg once daily for all subjects in cohort E from day 8 onwards (n = 5 received at least 1 dose of GLPG1205 150 mg; n = 2 discontinued on day 8 following 1 dose of GLPG1205 150 mg due to TEAEs). Two subjects in the GLPG1205 200‐mg once‐daily dose group, who had experienced a TEAE of dehydration, also showed abnormally high laboratory values for hematocrit, hemoglobin, and red blood cell count on day 8 of the study, which were considered clinically significant (for full details on TEAEs, see Table S2b). During the study, 4 subjects were observed with a treatment‐emergent abnormality during the physical examination (GLPG1205 50 mg once daily, n = 1; GLPG1205 100 mg once daily, n = 1; GLPG1205 200 mg once daily, n = 2); none of which were considered clinically significant and were therefore not reported as TEAEs.

#### Study 2

In part 1 of study 2, headache was the most commonly reported TEAE (n = 8; Table S3). All incidences of headache were rated as mild in intensity and were considered treatment related. One subject receiving placebo discontinued the study due to an AE (pain in extremity) having received 11 doses. In part 2 of the study (loading dose), the most commonly reported TEAE was nausea (Table S3; mild intensity, n = 2; moderate intensity, n = 1); 2 of these cases were considered treatment related. The total number of TEAEs was similar across age groups and between GLPG1205 dose groups (including the loading dose group) and placebo (Table S3). One clinically significant, treatment‐emergent physical examination abnormality was reported during the early discontinuation visit on day 18 (“pain left hip with endorotation”).

### Pharmacokinetic Profile

#### Study 1

**Figure 2:**
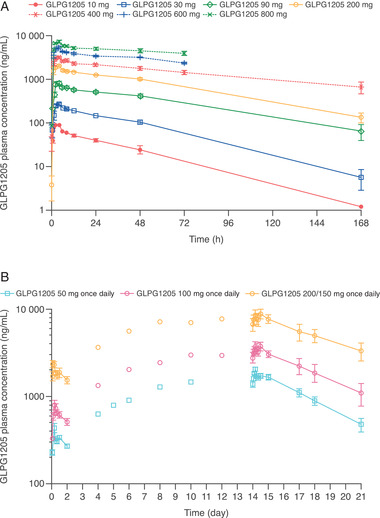
GLPG1205 plasma concentration vs time profiles for the (A) SAD and (B) MAD parts of study 1. No samples were collected at 168 hours after dosing for GLPG1205 600 and 800 mg. All data are mean ± standard error. MAD, multiple ascending doses; SAD, single ascending doses.

In the SAD part of study 1, mean plasma concentration‐time profiles (Figure 2A) and GLPG1205 plasma exposure (C_max_, AUC_0‐24h_, and AUC_0‐inf_; Table 4A) increased with increasing single doses of GLPG1205. GLPG1205 exposure did not markedly deviate from dose‐proportionality between 10 and 800 mg. After single administration, GLPG1205 was rapidly absorbed with a median t_max_ range of 2.0 to 4.0 hours, and was eliminated with a mean t_1/2,λz_ range of 30.1 to 140 hours (Table 4A).

**Table 4:** Summary of PK Parameters in Study 1 for the (A) SAD and (B) MAD Parts

A							
	GLPG1205						
PK Parameter	10 mg (n = 6)	30 mg (n = 6)	90 mg (n = 6)	200 mg (n = 6)	400 mg (n = 6)	600 mg (n = 6)	800 mg (n = 6)
C_max_ (ng/mL)	94.2 (15.8)	284 (6.65)	890 (14.0)	2200 (8.65)	3390 (14.0)	5530 (11.4)	7500 (13.5)
C_24h_ (ng/mL)	39.8 (23.3)	144 (8.96)	511 (14.0)	1260 (6.78)	2150 (16.3)	3410 (13.1)	5000 (18.6)
t_max_ (h)	2.0 (1.0–4.0)	4.0 (1.0–6.0)	4.0 (2.0–4.0)	3.0 (1.0–4.0)	4.0 (2.0–8.0)	4.0 (2.0–4.0)	4.0 (2.0–4.0)
AUC_0‐24h_ (μg • h/mL)	1.33 (13.5)	4.47 (5.92)	14.3 (12.5)	35.8 (4.94)	58.0 (13.9)	94.0 (12.5)	130 (13.6)
AUC_0–inf_ (μg • h/mL)	3.31 (49.0)^N = 3^	11.5 (21.8)^N = 4^	52.3 (35.8)^N = 4^	120 (16.0)^N = 4^	224 (NC)^N = 2^	NC^N = 0^ ^a^	NC^N = 0^ ^a^
t_1/2,λz_ (h)	32.2 (42.8)	30.1 (28.7)^N = 5^	57.7 (47.9)	54.3 (42.2)^N = 5^	92.4 (30.9)	75.0 (34.7)^N = 3^	140 (NC)^N = 2^

B						
	Day 1			Day 14		
PK Parameter	GLPG1205 50 mg Once Daily (n = 6)	GLPG1205 100 mg Once Daily (n = 6)	GLPG1205 200 mg Once Daily (n = 6)	GLPG1205 50 mg Once Daily (n = 6)	GLPG1205 100 mg Once Daily (n = 6)	GLPG1205^b^ 200/150 mg Once Daily (n = 3)
C_max_, ng/mL	469 (11.2)	875 (18.2)	2450 (20.1)	2050 (16.0)	3910 (28.2)	8900 (22.2)
t_max_, h	3.0 (2.0–4.0)	4.0 (2.0–6.0)	3.0 (2.0–4.0)	4.0 (2.0–4.0)	4.0 (2.0–12.0)	12.0 (4.0–12.0)
AUC_Τ_,^c^ μg • h/mL	7.58 (10.5)	14.4 (18.0)	42.9 (22.9)	41.3 (14.7)	82.7 (25.8)	195 (23.0)
R_ac_ ^d^	…	…	…	5.50 (0.98)	5.79 (1.10)	4.80 (0.68)
t_1/2,λz_, h	NC	NC	NC	76.7 (25.1)	88.6 (34.9)	141 (37.5)
Ae_(Τ)_, % dose	0.0785 (22.9)	0.0746 (30.1)^N = 4^	0.123 (27.0)	0.428 (22.0)^N = 5^	0.480 (27.2)	NC

Ae, cumulative amount of GLPG1205 excreted in urine; AUC_0‐inf_, area under the plasma concentration–time curve from time 0 to infinity; AUC_0‐24h_, area under the plasma concentration–time curve from time 0 to 24 hours; AUC_Τ_, area under the plasma concentration–time curve over the dosing interval; C_max_, maximum observed plasma concentration; C_24h_, plasma concentration at 24 hours after dosing; CV, coefficient of variation; MAD, multiple ascending doses; NC, not calculated; R_ac_, accumulation ratio; SAD, single ascending doses; t_max,_ time occurrence of maximum observed plasma concentration; t_1/2,λz_, apparent terminal half‐life.

Values are arithmetic means (coefficient of variation [CV%]) except median (minimum‐maximum) for t_max_. Ae_(Τ)_ was calculated over 24 hours. N = 6 unless otherwise indicated.

^a^ t_1/2_,_λz_ could not be estimated for 3 of 6 subjects treated with GLPG1205 600 mg and for 4 out of 6 subjects treated with GLPG1205 800 mg, resulting in no AUC_0‐inf_ value; the AUC_0‐inf_ for the other 3 and 2 subjects, respectively, had AUC extrapolations >20%.

^b^ In the 200‐mg once‐daily dose cohort, the dose was switched to 150 mg once daily as of day 8. Dose was normalized to a 150‐mg dose on day 14.

^c^ Dosing interval (24 hours).

^d^ R_ac_ was calculated with AUC_Τ_, and with GLPG1205 200 mg on day 1 and 150 mg on day 14.

In the MAD part of study 1, after once‐daily dosing for 14 days, steady‐state exposure (C_max_ and AUC_Τ_) to GLPG1205 increased in proportion with the dose from 50 to 100 mg once daily, with no observed change in absorption rate (Figure 2B; Table 4B). Steady state in GLPG1205 plasma concentrations was reached after 9 dosing days (by day 10), irrespective of dose, and accumulation ratios were similar between dosing groups (R_ac_ for GLPG1205 50 mg once daily, 100 mg once daily, and 200/150 mg once daily were 5.50, 5.79, and 4.80, respectively; Table 4B). The excretion of unchanged GLPG1205 in urine over 24 hours was <0.48% of the administered dose in 24 hours at steady state on day 14, and 57% to 93% of the total amount was excreted within the first 12 hours.

No significant day and dose effects were observed on 6β‐OH‐cortisol/cortisol ratio in urine following once‐daily dosing of GLPG1205 50 mg, 100 mg, and 200/150 mg once daily.

#### Study 2

**Figure 3:**
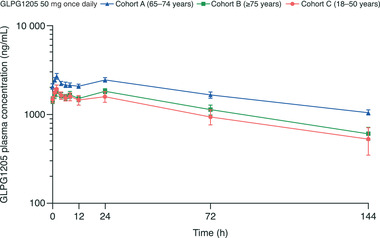
GLPG1205 plasma concentration vs time profiles for day 14 of the effect of aging cohorts. All data are mean ± standard error.

In part 1 of study 2, after once‐daily administration of GLPG1205 50 mg for 14 days, t_max_ was reached within 2 to 3 hours, and GLPG1205 plasma concentration plateaued up to 24 hours after dosing in all 3 age groups (Table 5A; Figure 3). Regardless of age, steady state was attained within 9 dosing days (by day 10), with accumulation ratios ranging from 5.02 to 6.16 (Table 5A). Significant age effects were observed for C_max_ (*P* = .0224), C_24h_ (*P* = .0164), t_max_ (*P* = .0210 at day 14), and AUC_0‐24h_ (*P* = .0072; Table 5A); pairwise comparisons showed there were no differences between the 3 age groups except for between the 65 to 74 years and 18 to 50 years age groups on day 14 for C_24h_ (*P* = .0089) and AUC_0‐24h_ (*P* = .0282).

**Table 5:** Summary of PK Parameters for (A) Part 1 (Effect of Age) and (B) Part 2 (Loading Dose) of Study 2

A								
	GLPG1205 50 mg Once Daily Cohort A, 65‐74 y (n = 6)		GLPG1205 50 mg Once Daily Cohort B, ≥75 y (n = 6)		GLPG1205 50 mg Once Daily Cohort C, 18‐50 y (n = 6)		ANOVA (*P* Value) Tukey's test	
PK Parameter	Day 1	Day 14	Day 1	Day 14	Day 1	Day 14	Age	Day
C_max_, μg/mL	0.557 (14.5)	2.75 (15.5)	0.440 (18.8)	1.96 (19.0)	0.476 (31.8)	1.95 (25.6)	0.0224	<.0001
C_24h_, μg/mL	0.334 (20.0)	2.44 (13.6)	0.290 (6.72)	1.82 (18.5)	0.301 (14.2)	1.59 (34.0)	0.0164	<.0001
AUC_0–24h,_ μg • h/mL	8.92 (19.5)	54.2 (14.0)	6.90 (11.5)	39.3 (18.8)	7.74 (18.6)	37.7 (27.4)	0.0072	<.0001
AUC_0–inf_, μg • h/mL	…	338 (26.0) ^N = 4^	…	236 (36.1) ^N = 4^	…	194 (71.7) ^N = 5^	0.1468	…
t_1/2,λz_, h	…	71.9 (24.4)	…	64.5 (28.8)	…	56.0 (43.9)	0.3233	…
R_ac_ ^a^	…	6.16 (0.74)	…	5.77 (1.34)	…	5.02 (1.66)	0.2111	…
t_max_, h^b^	2.0 (2.0‐4.0)	2.0 (1.0‐2.0)	2.0 (1.0‐6.0)	3.0 (2.0‐24.0)	2.0 (1.0‐4.0)	2.0 (2.0‐2.0)	Day 1: 0.9956 Day 14: 0.0210	.9102

B			
PK Parameter at Day 14	Cohort A, 65‐74 y GLPG1205 50 mg Once Daily (n = 6) Day 14	Cohort D, 65‐74 y GLPG1205 50 mg Once Daily With 250‐mg Loading Dose (n = 8) Day 14	ANOVA (*P* Value) With PE and 90% CI^c^ Cohort D vs Cohort A
C_max_, μg/mL	2.75 (15.5)	2.63 (17.6)	.6246
C_24h_, μg/mL	2.44 (13.6)	2.36 (19.5)	.6612
AUC_0–24h_, μg • h/mL	54.2 (14.0)	53.7 (18.3)	.8567
AUC_0–inf_, μg • h/mL	338 (26.0)^N = 4^	250 (28.4)^N = 6^	.1119
t_1/2,λz_, h	71.9 (24.4)	55.4 (16.8)^N = 7^	.0755
R_ac_	6.13 (11.6)	1.35 (22.8)	<.0001 22.11 (18.46‐26.48)
t_max_, h	2.0 (1.0–2.0)	2.0 (1.0–24.0)	…

AUC_0‐inf_, area under the plasma concentration–time curve from time 0 to infinity; AUC_0‐24h_, area under the plasma concentration–time curve from time 0 to 24 hours; CI, confidence interval; C_max_, maximum observed plasma concentration; C_24h_, plasma concentration at 24 hours after dosing; PK, pharmacokinetic; R_ac_, accumulation ratio; t_max,_ time occurrence of maximum observed plasma concentration; t_1/2,λz_, apparent terminal half‐life

Values are arithmetic mean (CV%) except median (minimum‐maximum) for t_max_.

^a^ R_ac_ was calculated with AUC_0‐24h_.

^b^ As t_max_ was a discrete variable dependent on selected blood sampling times, the effect of aging and of day were assessed using nonparametric tests (Kruskal‐Wallis's test and Wilcoxon's rank sum test for aging, and Wilcoxon's signed‐rank test for day).

^c^ Point estimate of the ratio in least square means together with its 95% CI.

In part 2 of study 2, following administration of the 250‐mg loading dose on day 1, GLPG1205 was quantifiable at the first sampling time point (1 hour after dosing) and maximal mean GLPG1205 concentration was reached at 12 hours after dosing. Steady state in GLPG1205 plasma concentration was reached after the loading dose of GLPG1205 250 mg (by day 2). When compared with the 14‐day GLPG1205 50‐mg once‐daily dose in cohort A, a GLPG1205 250‐mg loading dose followed by a 13‐day GLPG1205 50‐mg once‐daily dose showed no difference in steady‐state exposure (Table 5B). On day 14, following administration of GLPG1205 50 mg once daily for 13 days (from day 2 to day 14), t_max_ was reached at 2 hours after dosing (Table 5B).

GLPG1205 was quantifiable in plasma up to day 20 (ie, 144 hours after the last dose on day 14) in all subjects. By the follow‐up visit (day 35, 504 hours after the last dose on day 14), GLPG1205 levels were below the limit of quantification in 5 of 8 subjects; values ranged from 2.01 to 21.6 ng/mL in those subjects in whom GLPG1205 was still quantifiable.

### Pharmacodynamic Profile

In the SAD part of study 1, following a single administration of GLPG1205, dose‐dependent inhibition of ligand binding to GPR84 was observed for the 30‐ to 800‐mg doses compared with subjects receiving placebo (Figure S3A). The greatest mean percentage inhibition of ligand binding to GPR84 was observed at 4 hours after dosing for GLPG1205 800 mg (96.2%). The inhibitory effect was sustained over time; at 24 hours after dosing, mean GPR84 ligand‐binding inhibition ranged from 34.8% to 93.2% for GLPG1205 doses of between 30 and 800 mg vs 8.6% for placebo.

In the MAD part of study 1, similarly to the SAD part, a dose‐dependent inhibition of ligand binding to GPR84 was observed after a single administration of GLPG1205, versus placebo (day 1) (Figure S3B). The inhibition was maintained after multiple administrations of GLPG1205 50 mg once daily and 100 mg once daily (day 14) (Figure S3C). After multiple administrations (day 14), mean inhibition was observed before dosing on day 14 (24 hours after dosing on day 13) for GLPG1205 50 mg once daily (78.6%) and 100 mg once daily (83.5%) versus 4.6% for placebo. Inhibition of ligand binding to the GPR84 receptor was sustained over time; at 24 hours after dosing (day 15), 77.9% and 71.6% mean inhibition was observed for the GLPG1205 50‐mg and 100‐mg once‐daily doses, respectively, versus 21.1% for placebo. Inhibition of ligand binding to GPR84 was analyzed at day 14 for the 200/150‐mg once‐daily dosing regimen; however, due to the protocol violation of lowering the dose from day 8 onward and the small number of subjects remaining on day 14, the data are not presented.

### Pharmacokinetic/Pharmacodynamic Correlations

**Figure 4:**
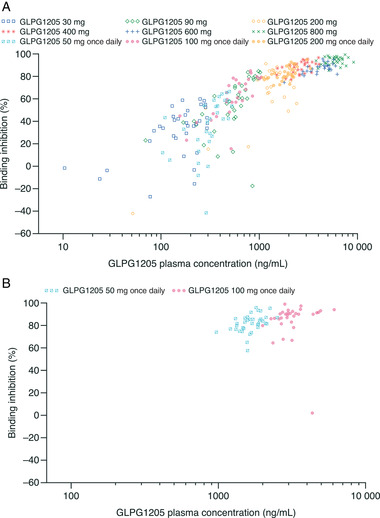
Correlation between percentage binding inhibition and GLPG1205 concentrations at (A) day 1 (SAD and MAD pooled) and (B) day 14 (MAD). MAD, multiple ascending doses; SAD, single ascending doses.

The percentage ligand binding inhibition increased with GLPG1205 plasma concentration, reaching almost complete inhibition and plateauing at ≈3000 ng/mL after a single administration on day 1 and at approximately 2000 ng/mL after multiple administrations on day 14 (Figure 4).

## Discussion

Study 1 demonstrated that single doses up to 800 mg once daily and multiple doses up to 100 mg once daily of GLPG1205 had favorable safety and tolerability profiles in healthy male subjects. Reduced tolerability was observed in the GLPG1205 200‐mg once‐daily dose cohort, with 3 subjects discontinuing study drug due to TEAEs including headache and nausea or vomiting. As a result, the dose was reduced to 150 mg on day 8 for the remainder of the study. As supported by safety and tolerability data from study 2, the maximum tolerated dose tested was GLPG1205 100 mg once daily.

PK results showed that exposure to GLPG1205 did not markedly deviate from dose‐proportionality from 10‐ to 800‐mg single doses. GLPG1205 was absorbed with a median t_max_ range of 2.0 to 4.0 hours, and was slowly eliminated. In both studies, once‐daily dosing for 14 days resulted in steady‐state being reached after 9 dosing days for all doses; overall accumulation ratios of between 4.77 and 5.71 (study 1, MAD) and between 4.81 and 6.13 (study 2, part 1) were observed, consistent with the long elimination half‐life of GLPG1205 (t_1/2,λz_ range, 76.7‐141 hours). The observed long elimination half‐life supports the use of the once‐daily dosing regimen in future clinical trials. Dosing levels to be tested in further trials will need to consider accumulation ratios, in line with a long elimination half‐life and dosing regimen, so as not to exceed safety margins. Steady‐state exposure (both C_max_ and AUC_T_) of GLPG1205 increased proportionally with the dose within the 50‐ to 100‐mg once‐daily dose range in study 1. Once‐daily dosing with GLPG1205 did not impact the 6β‐OH‐cortisol/cortisol ratio, which suggests that GLPG1205 likely does not interact with CYP3A4^16^; however, this finding requires confirmation via a clinical drug‐drug interaction with midazolam as an index‐sensitive CYP3A4 substrate. In study 2, administration of a GLPG1205 250 mg loading dose on day 1 followed by GLPG1205 50 mg once daily for 13 days, resulted in steady state being attained earlier by day 2 (ie, after the loading dose). Urine excretion was investigated over only 24 hours; thus, based on the long half‐life of GLPG1205, the amount of GLPG1205 excreted in urine may have been underestimated.

In study 2, exposure to GLPG1205 was similar in the 3 age groups following administration of GLPG1205 50 mg once daily for 14 days, suggesting that age has no effect on GLPG1205 exposure and there is no need for dose adjustments based on age. Administration of GLPG1205 50 mg once daily in all age groups, with or without a loading dose of 250 mg, did not reveal any safety concerns. A separate study has demonstrated that there is no food effect on GLPG1205 exposure (data on file at Galapagos).^17^

After a single administration of GLPG1205, GPR84 receptor occupancy measured as inhibition of ligand binding to GPR84 was observed for the 30‐ to 800‐mg doses compared with placebo with a concentration‐dependent effect. After 13 days of once‐daily GLPG1205 administration, ligand binding was already strongly inhibited before dosing for all doses. Based on the results from both the SAD and MAD parts of study 1, it can be concluded that GLPG1205 caused an extensive and sustained reduction in GPR84 receptor occupancy, suggesting that the 50‐mg dose of GLPG1205 may potentially be considered as one of the therapeutic doses in further clinical development.

The role of GPR84 in inflammatory and metabolic disease have been known for some time,^2^, ^3^, ^4^ but more recently a role in fibrotic disease has begun to emerge.^1^ In an adenine‐induced chronic kidney disease mouse model, deletion of *Gpr84* reduced kidney fibrosis compared with wild type, indicating a deleterious role for GPR84 in renal nephropathy.^1^ Furthermore, in a bleomycin‐induced mouse model of pulmonary fibrosis, treatment with PBI‐4050, described as a GPR84 modulator and GPR40 agonist, caused a 47% reduction of histological lesions in the lung (compared with vehicle),^1^ supporting a role for GPR84 in fibrotic disease. Whether GPR84 modulators can protect against fibrotic disease in human remains to be investigated.

The small sample sizes and homogeneity (eg, race, gender) of subjects were limitations of the studies; however, the sample sizes and characteristics were appropriate for phase 1 studies in healthy subjects. Other limitations include the potential underestimation of urine excretion due to duration of investigation as previously discussed, and length of blood sampling in the MAD part of study 1.

## Conclusions

In conclusion, single (up to 800 mg) and multiple (up to 100 mg once daily) ascending oral doses of GLPG1205, administered to healthy male subjects, had favorable safety, tolerability, and PK/PD profiles and resulted in effective inhibition of GPR84 binding. Age had no effect on the PK of multiple doses of GLPG1205.

Furthermore, a single GLPG1205 250‐mg loading dose resulted in reduced time to reach steady‐state exposure comparable with the 50‐mg once‐daily dose, from 9 dosing days to 1 day. These results support the further clinical development of GLPG1205 with once‐daily dosing.

## Conflicts of Interest

S.D., E.H., L.M., E.S., H.T., T.V.K., and S.H. are employees of Galapagos. J.D. and J.B. are former employees of Galapagos.

## Funding

The study and writing/editorial support were funded by Galapagos, Mechelen, Belgium.

## Author Contributions

All authors provided substantial contribution to the design or the acquisition, analysis, or interpretation of data. Authors critically reviewed the article for important intellectual content. All authors read and approved the final version of the article.

## Supporting information



Additional supplemental information can be found by clicking the Supplements link in the PDF toolbar or the Supplemental Information section at the end of the web‐based version of this article.

## Data Availability

Data will not be shared.
