# Supplementary material for: GLPG1205, a GPR84 Modulator: Safety, Pharmacokinetics, and Pharmacodynamics in Healthy Subjects
Source: Clin Pharmacol Drug Dev. 2021 May 7;10(9):994–1006. doi: 10.1002/cpdd.955 (PMC8453901; doi:10.1002/cpdd.955)
Supplement: Supplementary file 1 — Additional supplemental information can be found by clicking the Supplements link in the PDF toolbar or the Supplemental Information section at the end of the web‐based version of this article. [file CPDD-10-994-s001.docx]

**Supplementary Table 1.** Exclusion criteria for the first-in-human and effect of aging and loading dose studies.

| **First-in-human study (study 1)** | **Effect of aging and loading dose study (study 2)** |
| --- | --- |
| Known hypersensitivity to study drug ingredients or a significant allergic reaction to any drug as determined by the investigator, such as anaphylaxis requiring hospitalization | Known hypersensitivity to GLPG1205 or excipients of the formulation. A history of significant allergic reaction to any drug, such as anaphylaxis requiring hospitalization |
| Positive serology for HBsAg or HCV | Positive serology for HBsAg, HCV or HIV |
| Any history of hepatitis from any cause with the exception of hepatitis A | Clinically significant illness in the 12 weeks prior to study screening |
| History of or a current immunosuppressive condition (e.g., HIV infection) | Current or sequelae of gastrointestinal, liver or kidney disease or any other condition that might interfere with absorption, distribution, metabolism or excretion of drugs |
| Symptoms of clinically significant illness in the 3 months before the initial study drug administration | History of malignancy in the last 5 years, except basal cell carcinoma of the skin that had been treated and with no evidence of recurrence |
| Presence or having sequelae of gastrointestinal, liver or kidney (creatinine clearance ≤80 mL/min using the Cockcroft formula; if calculated result ≤80 mL/min, a 24-hour urine collection to determine actual value can be done) or other conditions known to interfere with the absorption, distribution, metabolism, or excretion of drugs | Clinically significant abnormalities detected on laboratory safety testing including: hemoglobin (<10 g/dL), white blood count (<3.0 x 10^9^ cells/L), neutrophil count (<1.5 x 10^9^ cells/L), platelet count (<100 x 10^9^ cells/L), serum ALT or AST (>2x ULN), and total bilirubin level (>1.5x ULN [except in cases of Gilbert’s syndrome]) |
| History of malignancy within the past 5 years (except for basal cell carcinoma of the skin that has been treated with no evidence of recurrence) | Clinically significant abnormalities detected on physical examination or vital signs considered relevant by the Investigator which the Investigator or designee considered should exclude the subject |
| Clinically significant abnormalities detected on ECG regarding either rhythm or conduction (e.g., QTcF ≥450 ms, or a known long QT syndrome), excluding first-degree heart block | Clinically significant abnormalities on ECG of rhythm or conduction (e.g., QTcF >450 ms, known long-QT syndrome), excluding first-degree heart block |
| Clinically significant abnormalities detected on vital signs | Active drug or alcohol abuse within 2 years prior to study screening |
| Significant blood loss (including blood donation [>450 mL]), or had a transfusion of any blood product within 12 weeks prior to the initial study drug administration | Significant blood loss, including blood donation of >450 mL, or having received a blood transfusion or blood product in the 12 weeks prior to study screening |
| Hemoglobin level below 12 g/dL | Consumption of large quantities of caffeinated coffee or tea (>6 cups/day), or equivalent. |
| Treatment with any drug known to have a well-defined potential for toxicity to a major organ in the last 3 months preceding the initial study drug administration | Concurrent or recent participation (within 4 weeks or 5 half-lives of the drug, whichever was longer) prior to screening in an investigational medicinal research study |
| Active drug or alcohol abuse (more than 3 glasses of wine or beer or equivalent/day) within 2 years prior to the initial study drug administration | A member of staff who was directly involved in the conduct of the study or a relative thereof |
| Consumption of a large quantity of coffee, tea (>6 cups per day) or equivalent | Any condition or circumstances that in the opinion of the Investigator may make a subject unlikely or unable to complete the study or comply with study procedures and requirements |
| Administration of an injectable drug within 30 days prior to the initial study drug administration, with the exception of local anesthetics |  |
| Concurrent participation or participation within 8 weeks prior to the initial study drug administration in a drug/device or biologic investigational research study |  |
| Investigator or any sub-investigator, research assistant, pharmacist, study coordinator, or other staff or relative thereof who is directly involved in the conduct of the study |  |
| Any condition or circumstances that in the opinion of the investigator could make a subject unlikely or unable to complete the study or comply with study procedures and requirements |  |
| Current child wish; a contraception method had to be used during the study and until 3 months after the end of the study. No sperm donation was allowed during the study and until 3 months after last study visit |  |

ECG, electrocardiogram; HBsAg, hepatitis B virus surface antigen; HCV, hepatitis C virus; HIV, human immunodeficiency virus; QTcF, corrected QT interval by Fredericia; ULN, upper limit of normal

**Supplementary Table 2.** All treatment-emergent adverse events in study 1 in the (A) SAD and (B) MAD study populations.

**A**

| **System Organ Class**  **Preferred Term** | **Placebo** | **GLPG1205** | | | | | | |
| --- | --- | --- | --- | --- | --- | --- | --- | --- |
|  | **Pooled**  **(n = 14)** | **10 mg (n = 6)** | **30 mg (n = 6)** | **90 mg (n = 6)** | **200 mg (n = 6)** | **400 mg (n = 6)** | **600 mg (n = 6)** | **800 mg (n = 6)** |
| Any TEAE | 0 | 2 (33.3) | 1 (16.7) | 0 | 1 (16.7) | 3 (50.0) | 0 | 5 (83.3) |
| Nervous system disorders | 0 | 1 (16.7) | 1 (16.7) | 0 | 0 | 1 (16.7) | 0 | 3 (50.0) |
| Headache | 0 | 1 (16.7) | 0 | 0 | 0 | 1 (16.7) | 0 | 1 (16.7) |
| Somnolence | 0 | 0 | 1 (16.7) | 0 | 0 | 0 | 0 | 1 (16.7) |
| Dizziness | 0 | 0 | 0 | 0 | 0 | 0 | 0 | 1 (16.7) |
| Gastrointestinal disorders | 0 | 0 | 0 | 0 | 1 (16.7) | 1 (16.7) | 0 | 2 (33.3) |
| Nausea | 0 | 0 | 0 | 0 | 1 (16.7) | 1 (16.7) | 0 | 2 (33.3) |
| Respiratory, thoracic, and mediastinal disorders | 0 | 0 | 0 | 0 | 0 | 0 | 0 | 2 (33.3) |
| Oropharyngeal pain | 0 | 0 | 0 | 0 | 0 | 0 | 0 | 2 (33.3) |
| Vascular disorders | 0 | 0 | 0 | 0 | 0 | 1 (16.7) | 0 | 0 |
| Hot flush | 0 | 0 | 0 | 0 | 0 | 1 (16.7) | 0 | 0 |
| Musculoskeletal and connective tissue disorders | 0 | 0 | 0 | 0 | 0 | 1 (16.7) | 0 | 0 |
| Torticollis | 0 | 0 | 0 | 0 | 0 | 1 (16.7) | 0 | 0 |
| Infections and infestations | 0 | 1 (16.7) | 0 | 0 | 0 | 0 | 0 | 0 |
| Urethritis chlamydial | 0 | 1 (16.7) | 0 | 0 | 0 | 0 | 0 | 0 |

**B**

| **System Organ Class**  **Preferred Term** | **Placebo** | **GLPG1205** | | |
| --- | --- | --- | --- | --- |
|  | **Pooled (n = 6)** | **50 mg QD (n = 6)** | **100 mg QD (n = 6)** | **200 mg QD (n = 6)** |
| Any TEAE | 2 (33.3) | 2 (33.3) | 5 (83.3) | 5 (83.3) |
| Nervous system disorders | 1 (16.7) | 0 | 3 (50.0) | 5 (83.3) |
| Headache | 1 (16.7) | 0 | 3 (50.0) | 4 (66.7) |
| Somnolence | 0 | 0 | 0 | 1 (16.7) |
| Dizziness | 0 | 0 | 0 | 1 (16.7) |
| Disturbance in attention | 0 | 0 | 0 | 1 (16.7) |
| Gastrointestinal disorders | 1 (16.7) | 1 (16.7) | 3 (50.0) | 4 (66.7) |
| Vomiting | 0 | 0 | 0 | 2 (33.3) |
| Nausea | 1 (16.7) | 0 | 1 (16.7) | 2 (33.3) |
| Diarrhea | 0 | 1 (16.7) | 2 (33.3) | 1 (16.7) |
| Flatulence | 0 | 0 | 0 | 1 (16.7) |
| Abdominal pain upper | 0 | 0 | 1 (16.7) | 0 |
| Abdominal pain | 0 | 0 | 0 | 1 (16.7) |
| Abdominal distension | 0 | 0 | 0 | 1 (16.7) |
| Abdominal discomfort | 0 | 0 | 1 (16.7) | 1 (16.7) |
| General disorders and administration site conditions | 0 | 0 | 0 | 3 (50.0) |
| Fatigue | 0 | 0 | 0 | 3 (50.0) |
| Skin and subcutaneous tissue disorders | 0 | 1 (16.7) | 2 (33.3) | 0 |
| Erythema | 0 | 0 | 1 (16.7) | 0 |
| Ecchymosis | 0 | 0 | 1 (16.7) | 0 |
| Dry skin | 0 | 1 (16.7) | 0 | 0 |
| Respiratory, thoracic and mediastinal disorders | 0 | 0 | 1 (16.7) | 2 (33.3) |
| Oropharyngeal pain | 0 | 0 | 1 (16.7) | 2 (33.3) |
| Metabolism and nutrition disorders | 0 | 0 | 0 | 2 (33.3) |
| Dehydration | 0 | 0 | 0 | 2 (33.3) |
| Decreased appetite | 0 | 0 | 0 | 1 (16.7) |
| Vascular disorders | 0 | 0 | 0 | 1 (16.7) |
| Hot flush | 0 | 0 | 0 | 1 (16.7) |
| Musculoskeletal and connective tissue disorders | 0 | 0 | 0 | 1 (16.7) |
| Musculoskeletal stiffness | 0 | 0 | 0 | 1 (16.7) |

Values are number of subjects with a given TEAE (%). All adverse events starting on or after first dosing are considered treatment-emergent adverse events. A subject could have experienced >1 TEAE of the same preferred term.

MAD, multiple ascending doses; QD, once daily; SAD, single ascending doses;
TEAE, treatment-emergent adverse event

**Supplementary Table 3.** All treatment-emergent adverse events in study 2.

|  | **Part 1** | | | | **Part 2, open label** | |
| --- | --- | --- | --- | --- | --- | --- |
| **System Organ Class**  **Preferred Term** | **Pooled placebo (n = 6)** | **GLPG1205  50 mg QD 65–74 years (n = 6)** | **GLPG1205  50 mg QD**  **≥ 75 years (n = 6)** | **GLPG1205  50 mg QD**  **18–50 years**  **(n = 6)** | **GLPG1205  250 mg loading dose + 50 mg QD 65–74 years**  **(n = 8)** | |
| Any TEAE | 5 (83.3) | 5 (83.3) | 3 (50.0)  1 (16.7)  0  0  0  0  0  0  0 | 5 (83.3)  2 (33.3)  2 (33.3)  0  0  3 (50.0)  3 (50.0)  1 (16.7)  1 (16.7) | 7 (87.5)  4 (50.0)  3 (37.5)  1 (12.5)  2 (25.0)  1 (12.5)  1 (12.5)  0  0  0  0 |  |
| Gastrointestinal disorders | 2 (33.3) | 1 (16.7)  0  0  0 |  |  |  |  |
| Nausea | 0 |  |  |  |  |  |
| Diarrhea | 1 (16.7) |  |  |  |  |  |
| Vomiting | 0 |  |  |  |  |  |
| Nervous system disorders | 2 (33.3)  2 (33.3)  1 (16.7) | 4 (66.7)  3 (50.0)  3 (50.0)  2 (33.3) |  |  |  |  |
| Headache |  |  |  |  |  |  |
| Infections and infestations |  |  |  |  |  |  |
| Nasopharyngitis | 0 |  |  |  |  |  |
| Renal and urinary disorders | 0 | 0  0 | 2 (33.3)  2 (33.3) | 0  0 |  |  |
| Polyuria | 0 |  |  |  |  |  |
| Reproductive system and breast disorders | 0 | 0 | 0 | 2 (33.3) | 0 |  |
| Erectile dysfunction | 0 | 0 | 0 | 2 (33.3) | 0 |  |

Values are number of subjects with a given TEAE (%). All adverse events starting on or after first dosing are considered treatment-emergent adverse events. A subject could have experienced > 1 TEAE of the same preferred term.

QD, once daily; TEAE, treatment-emergent adverse event

**Supplementary Figure 1.** Subject disposition in the (A) SAD and (B) MAD parts of study 1.

G, GLPG1205; MAD, multiple ascending doses; QD, once daily; SAD, single ascending doses; TEAE, treatment-emergent adverse event

**A**

**
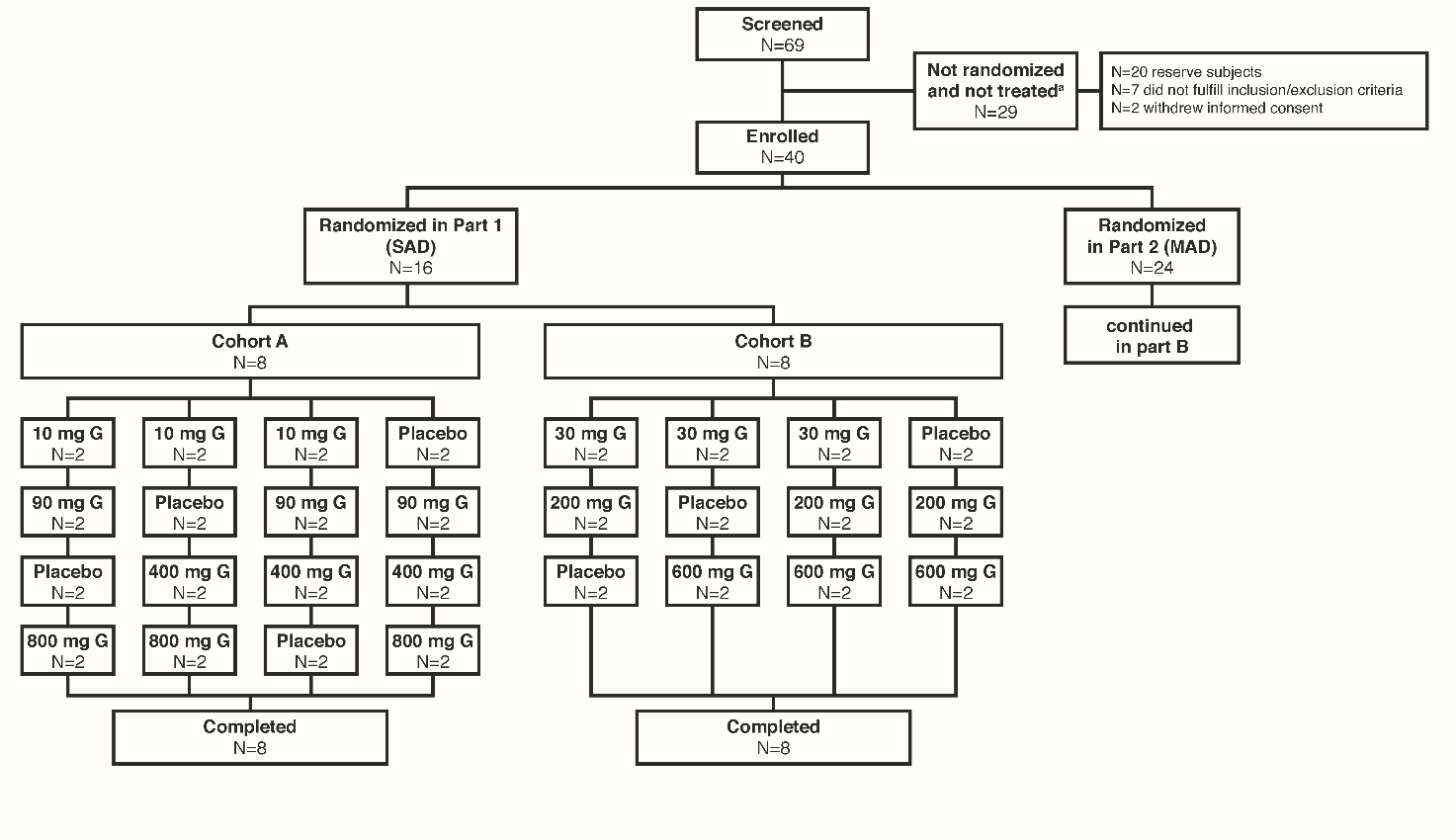
**

**B**


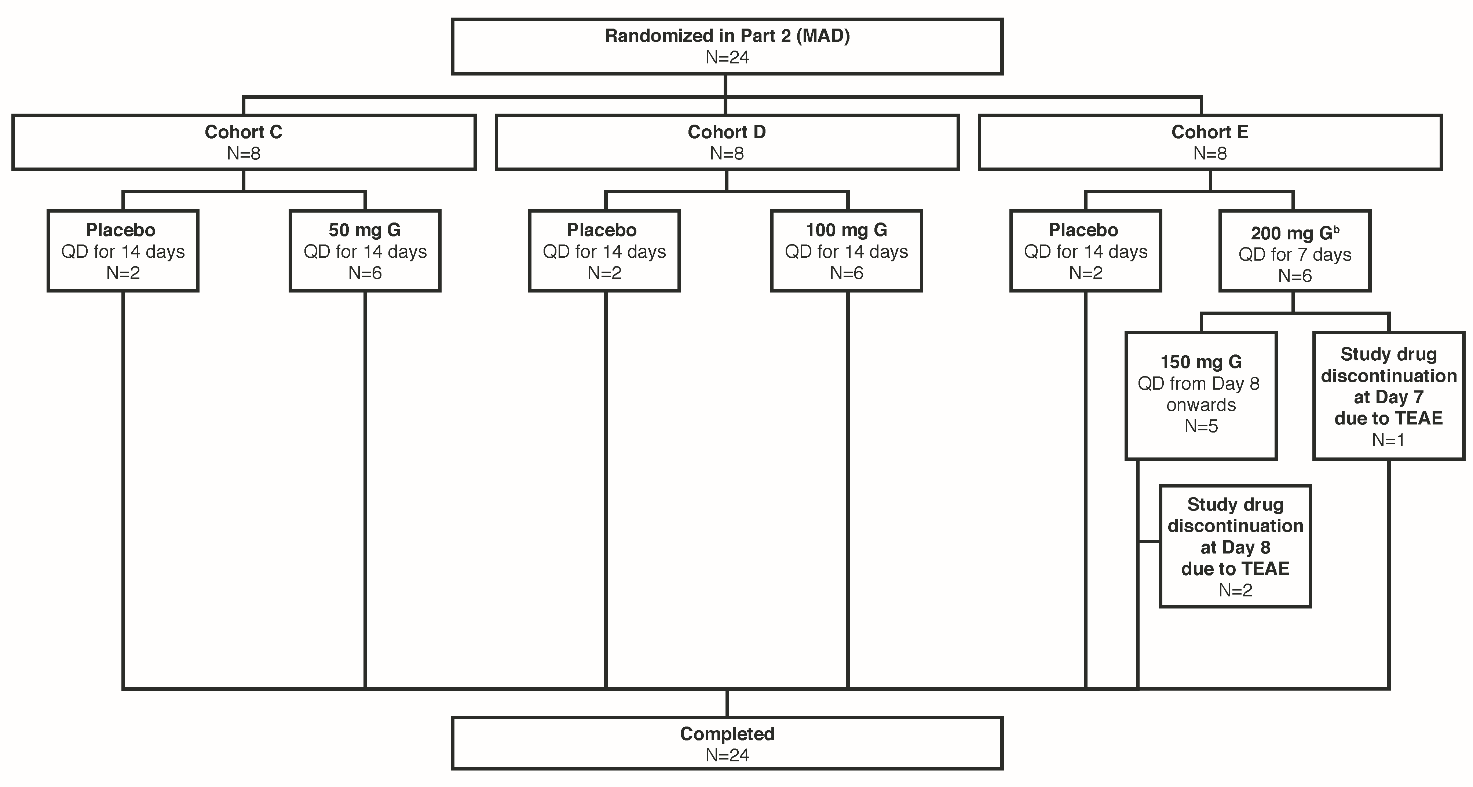


^a^ After database lock the investigator stated that 2 of the 20 reserve subjects withdrew consent and one no longer met the inclusion/exclusion criteria

^b^ Subjects in the 200 mg GLPG1205/placebo dose group switched to 150 mg GLPG1205/placebo as of day 8 (Subject 217 had the last dose of study drug at day 7, Subjects 218 and 223 on day 8 [150 mg]); all subjects completed the (remaining) study visits as planned per protocol

**Supplementary Figure 2.** Subject disposition in the effect of aging study.

AE, adverse event

**
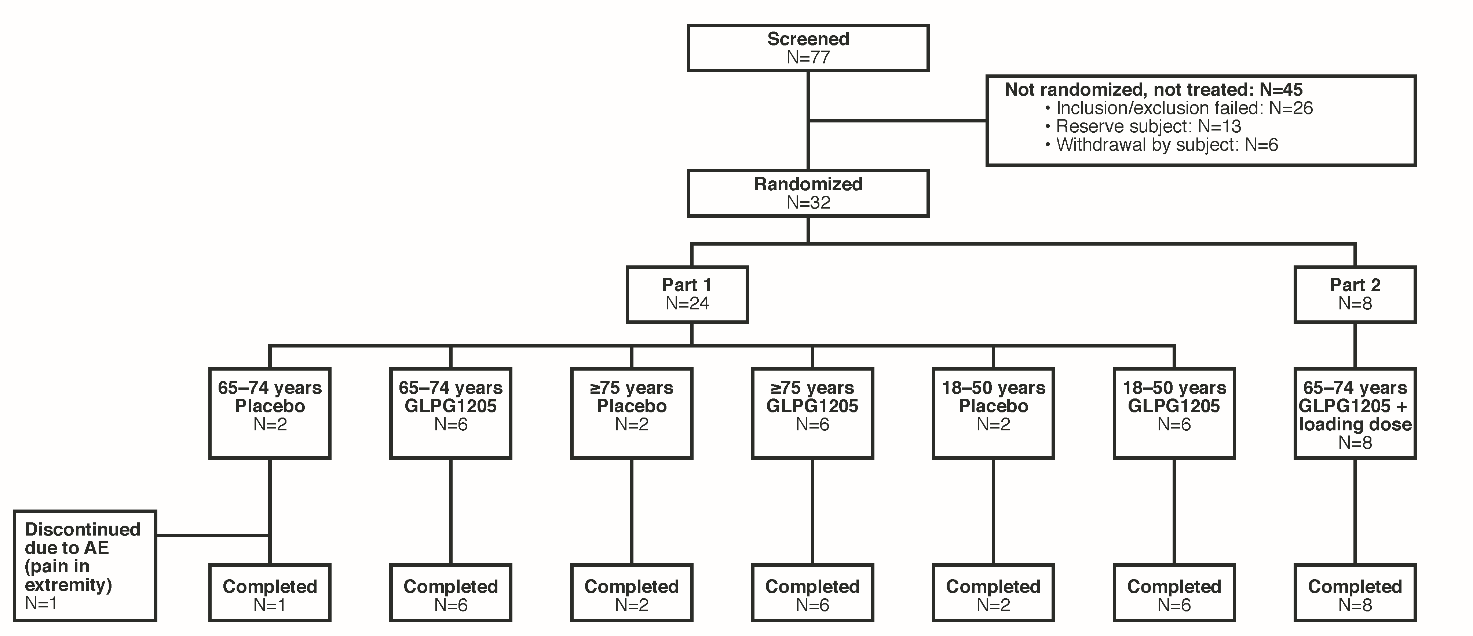
**

**Supplementary Figure 3.** Percentage binding inhibition of the tritiated ligand to the receptor GPR84 versus baseline for the (A) SAD part, (B) MAD part on day 1, and (C) MAD part on day 14. In (C), due to the dose adaptation from GLPG1205 200 to 150 mg QD on day 8, only data from day 1 is presented in the graph. All data are mean ± SE

MAD, multiple ascending doses; QD, once daily; SAD, single ascending doses; SE, standard error

**A**

**
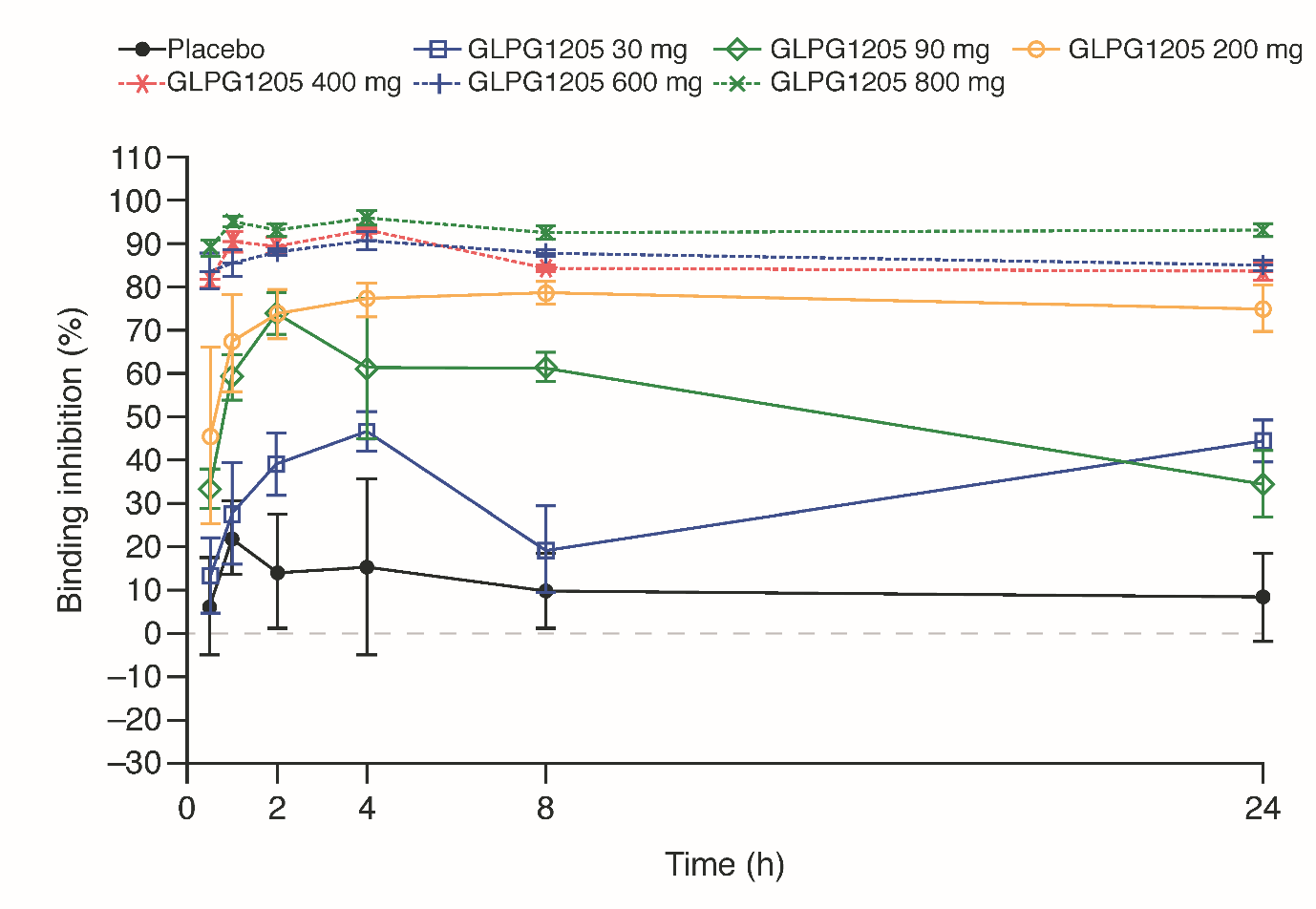
**

**B**

**
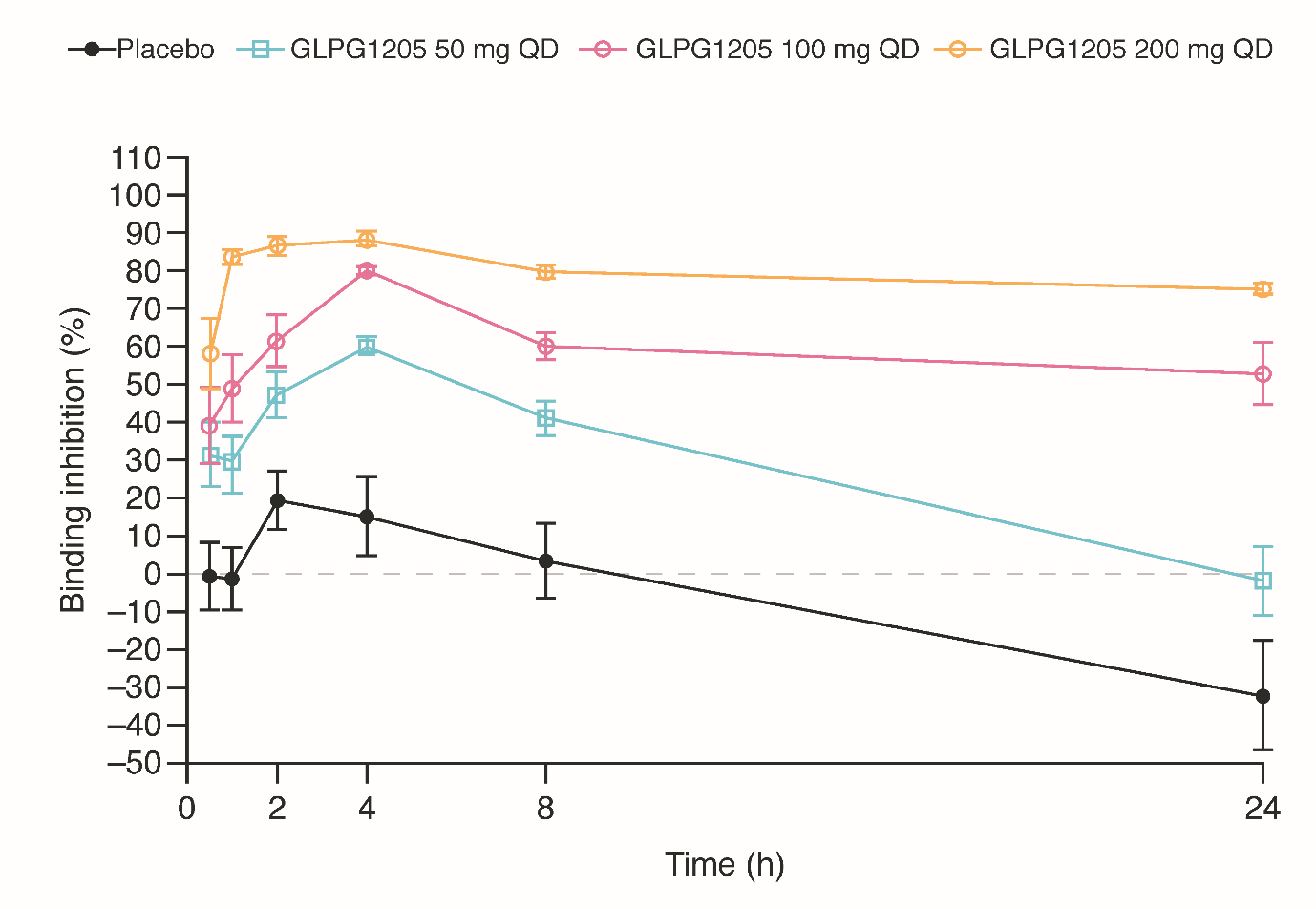
**

**C**

**
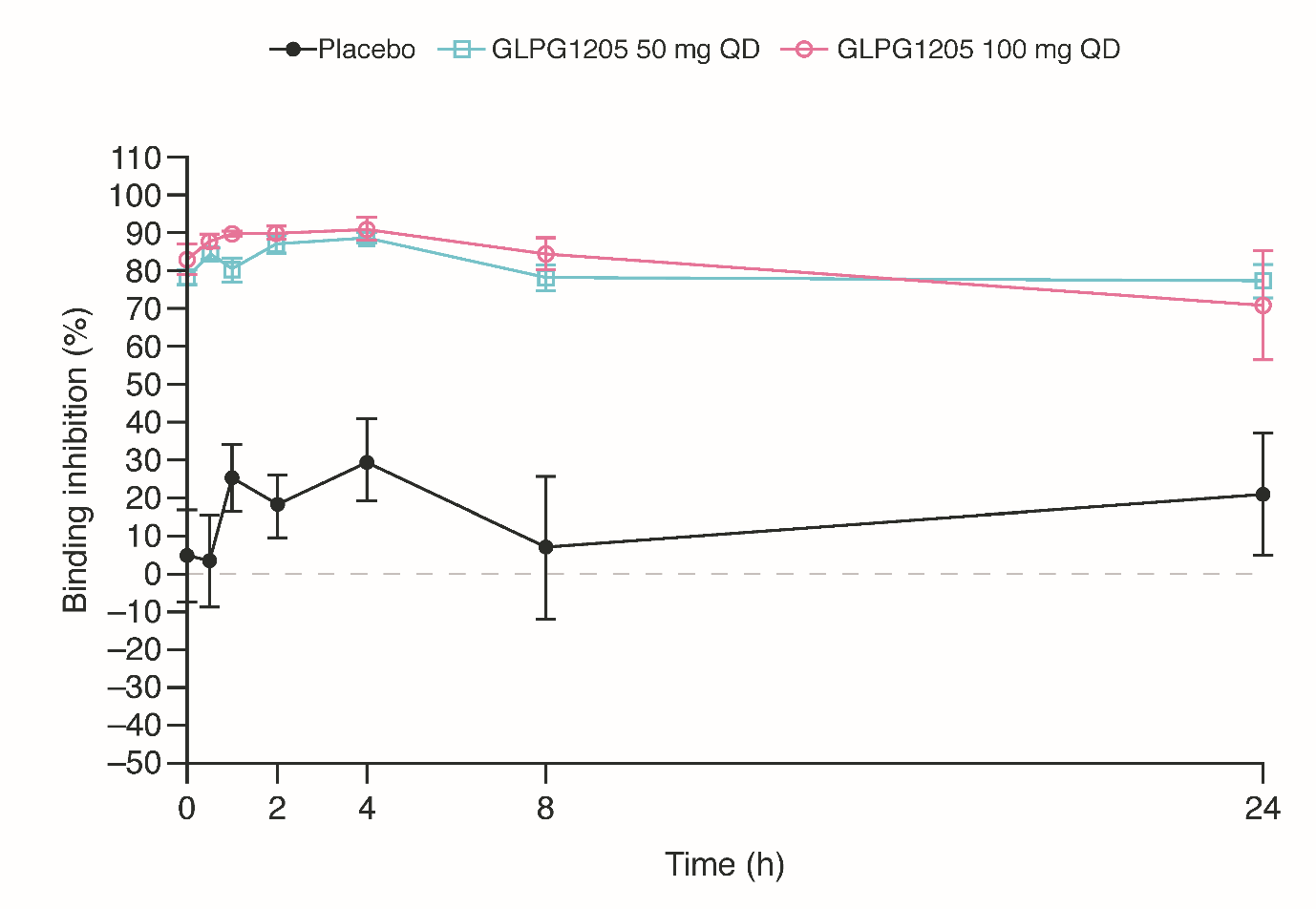
**
